# Adjuvant Chemoradiotherapy or Chemotherapy After D2 Gastrectomy in Gastric Cancer: A Randomized Clinical Trial

**DOI:** 10.1001/jamanetworkopen.2026.16154

**Published:** 2026-06-15

**Authors:** Xin Wang, Ouying Yan, Jitao Zhou, Feng Wen, Yali Shen, Zhiping Li, Qiu Li, Wenling Wang, Xiaohong Cai, Shisheng Tan, Zi Wang, Qing Qiao, Dan Cao, Meng Qiu, Jiyan Liu, Hongfeng Gou, Ming Liu, Cheng Yi, Yu Yang, Qing Zhu, Deyun Luo, Yaqin Zhao, Ye Chen, Shang Wang, Qiaoli Wang, Ke Cheng, Min Ren, Xin Liu, Feng Xu, Feng Bi

**Affiliations:** 1 Division of Abdominal Tumor Multimodality Treatment, West China Hospital, Sichuan University, Sichuan, China; 2 State Key Laboratory of Biotherapy, West China Hospital, Sichuan University, Sichuan, China; 3 Department of Radiation Oncology, West China Hospital, Sichuan University, Sichuan, China; 4 Department of Abdominal Oncology, Affiliated Cancer Hospital of Guizhou Medical University, Guiyang, Guizhou, China; 5 Department of Medical Oncology, Sichuan Cancer Hospital and Institute, Chengdu, Sichuan, China; 6 Department of Oncology, Guizhou Provincial People's Hospital, Guiyang, Guizhou, China; 7 Department of Oncology, Leshan People's Hospital, Leshan, Sichuan, China; 8 Department of Colorectal Cancer Center, West China Hospital, Sichuan University, Chengdu, Sichuan, China; 9 Department of Biotherapy and Cancer Center, State Key Laboratory of Biotherapy, West China Hospital, Sichuan University, Chengdu, Sichuan, China; 10 Gastric Cancer Center, Division of Medical Oncology, Cancer Center, West China Hospital, Chengdu, Sichuan, China; 11 Lung Cancer Center, West China Hospital, Sichuan University, Chengdu, Sichuan, China

## Abstract

**Importance:**

Despite advances in D2 gastrectomy (ie, gastrectomy with D2 lymphadenectomy) and adjuvant chemotherapy, the value of adding postoperative radiotherapy (RT) to adjuvant chemotherapy after D2 gastrectomy remains a topic of ongoing debate.

**Objective:**

To assess whether the addition of RT to the S-1 plus oxaliplatin (SOX) chemotherapy regimen increases disease-free survival (DFS) in patients with T4 or node-positive gastric cancer after D2 gastrectomy.

**Design, Setting, and Participants:**

This open-label, phase 3 randomized clinical trial was conducted between December 1, 2012, and August 30, 2022, at 5 large tertiary hospitals in China. Eligible patients were aged 18 to 70 years with gastric adenocarcinoma who underwent R0 resection with D2 lymphadenectomy, had pathologic stage T4 or node-positive disease, and had no evidence of metastasis (M0). Intention-to-treat statistical analyses were conducted from January 14 to March 31, 2025.

**Interventions:**

Participants were assigned 1:1 to receive concurrent chemoradiotherapy (SOX RT) or chemotherapy alone (SOX). The SOX RT group received 1 cycle of induction SOX, followed by RT (50.4 Gy in 28 fractions) with concurrent S-1 (50 mg twice daily), and then 3 cycles of SOX. The SOX group received 6 cycles of SOX (S-1 30-40 mg/m^2^ twice daily on days 1-14; oxaliplatin 130 mg/m^2^ on day 1, every 3 weeks).

**Main Outcomes and Measures:**

The primary end point was 3-year DFS. Secondary end points included overall survival (OS) and adverse events.

**Results:**

A total of 620 patients were randomly assigned to the SOX group (n = 311) or SOX RT group (n = 309). Patients had a median (IQR) age of 55 (47-62) years and included 401 males (64.7%). Overall, 274 patients (44.2%) had T4 disease, and 590 (95.2%) had node-positive disease. In the primary analysis for the prespecified 3-year DFS end point, there was no evidence of a between-group difference in DFS (SOX RT vs SOX: HR, 0.98; 95% CI, 0.73-1.33). Similarly, there was no evidence of a between-group difference in OS (HR, 0.86; 95% CI, 0.60-1.23). The Kaplan-Meier–estimated 3-year DFS rates were 70.5% in the SOX RT group and 69.3% in the SOX group (log-rank *P* = .93), and the corresponding 3-year OS rates were 80.8% and 78.4%, respectively (log-rank *P* = .41). At 5 years, the DFS rates were 60.0% and 57.3% (log-rank *P* = .76) and the corresponding OS rates were 73.7% and 71.4% (log-rank *P* = .55) in the SOX RT and SOX groups, respectively. Treatment-related adverse events were similar between groups.

**Conclusions and Relevance:**

In this randomized clinical trial of patients with T4 or node-positive gastric cancer who underwent D2 gastrectomy, SOX RT did not significantly improve DFS or OS. Accordingly, among patients with D2-resected gastric cancer, the findings do not support routine addition of adjuvant chemoradiotherapy to chemotherapy.

**Trial Registration:**

Chinese Clinical Trial Registry Identifier: ChiCTR-TRC-12002919

## Key Points

**Question:**

Is adjuvant chemoradiotherapy more effective than chemotherapy alone in patients with gastric cancer and T4 or node-positive disease following D2 gastrectomy (ie, gastrectomy with D2 lymphadenectomy)?

**Findings:**

In this randomized clinical trial involving 620 patients, adding radiotherapy to adjuvant S-1 plus oxaliplatin (SOX) chemotherapy after D2 gastrectomy did not significantly improve disease-free survival or overall survival compared with SOX alone.

**Meaning:**

These findings suggest that routine intensification with postoperative radiotherapy added to adjuvant SOX may not be warranted after D2 R0 resection for T4 or node-positive gastric cancer.

## Teaser

This randomized clinical trial evaluates whether the addition of radiotherapy to adjuvant S-1 plus oxaliplatin chemotherapy increases disease-free survival among patients undergoing D2 gastrectomy for T4 or node-positive gastric cancer.

## Introduction

Gastric cancer is a prevalent malignant neoplasm with high incidence and mortality worldwide. It is characterized by a subtle onset, an aggressive course, and substantial biological variability, all of which pose considerable challenges to effective treatment.^1,2^ Although surgery remains the cornerstone of curative therapy, a substantial proportion of patients experience recurrence.^3,4^ The US-based Intergroup-0116 study supported concurrent chemoradiation therapy (CRT) as an adjuvant treatment, reporting significant improvements in overall survival (OS) (hazard ratio [HR], 1.32; *P* = .005) and relapse-free survival (HR, 1.51; *P* = .001) after 10 years of follow-up compared with surgery alone.^5^ However, only 10% of patients in the Intergroup-0116 study underwent D2 lymph node dissection. Long-term follow-up studies have found that D2 gastrectomy (ie, gastrectomy with D2 lymphadenectomy) can reduce gastric cancer–specific mortality.^6,7,8^ As a result, treatment guidelines now recommend D2 gastrectomy for patients with resectable gastric cancer.^9^ Despite these advances, adding adjuvant radiotherapy (RT) to the chemotherapy regimen after D2 lymph node dissection remains a topic of ongoing debate.

Several clinical studies in Asian countries have explored optimal adjuvant therapy strategies for patients following D2 gastrectomy. The ACTS-GC (Adjuvant Chemotherapy Trial of TS-1 for Gastric Cancer) and CLASSIC (Capecitabine and Oxaliplatin Adjuvant Study in Stomach Cancer) trials demonstrated that adjuvant chemotherapy significantly improved survival compared with surgery alone in patients with gastric cancer after D2 lymph node dissection.^8,10,11^ The ARTIST trial further evaluated the addition of RT to chemotherapy after D2 gastrectomy to assess its impact on survival. A subgroup analysis suggested that adjuvant RT may improve disease-free survival (DFS) in patients with lymph node–positive cancer after D2 resection.^7^ Subsequently, the ARTIST 2 trial, which was conducted in South Korea and was terminated early, indicated no significant difference in efficacy between chemotherapy alone using S-1 plus oxaliplatin (SOX) and concurrent CRT after D2 gastrectomy. Subgroup analyses also revealed no statistically significant differences.^12^ At West China Hospital of Sichuan University, phase 1 or 2 trials investigated the leucovorin, fluorouracil, and oxaliplatin (FOLFOX) regimen combined with concurrent RT (50.4 Gy) after D2 gastrectomy. These studies demonstrated the efficacy and safety of this treatment approach.^13^ The phase 2 study showed OS rates of 95% at 1 year and 82% at 2 years and DFS rates of 85% at 1 year and 78% at 2 years among patients who received FOLFOX combined with concurrent RT following D2 surgery. Additionally, a phase 1 study of the SOX regimen combined with concurrent RT determined the maximum tolerated dose and safety profile of S-1 during concurrent RT.^14^

The role of RT after D2 gastrectomy warrants further investigation. Building on the phase 1 or 2 trials conducted at West China Hospital of Sichuan University, we designed this randomized phase 3 study to assess whether the addition of RT to the SOX chemotherapy regimen increases DFS in patients with T4 or node-positive gastric cancer after D2 gastrectomy.

## Methods

### Study Design

This open-label, multicenter, investigator-initiated randomized clinical trial was conducted between December 1, 2012, and August 30, 2022, at 5 large tertiary hospitals in China. The institutional review boards at each participating center approved the study protocol (Supplement 1), which adhered to the ethical principles of the Declaration of Helsinki^15^ and local guidelines. Written informed consent was obtained from all patients before randomization. Trial coordination was supported by West China Hospital of Sichuan University in Chengdu, China. We followed the Consolidated Standards of Reporting Trials (CONSORT) reporting guideline.

### Study Procedures

We used a computer-generated randomization sequence for participant allocation. To ensure balanced treatment assignment across disease stages, stratified block randomization was implemented, with disease stage as the stratification factor. Sequential numbering was used to ensure allocation concealment. Patients were identified for enrollment by their oncologists and randomly assigned in a 1:1 ratio to either the concurrent CRT arm (SOX RT) or the chemotherapy-alone arm (SOX).

In the SOX RT arm, patients received 1 cycle of induction chemotherapy with the SOX regimen 21 days before RT. RT was administered at a total dose of 50.4 Gy in 28 fractions (1.8 Gy/d, 5 days per week), using 3-dimensional conformal RT (3D-CRT) and intensity-modulated RT (IMRT) or volumetric modulated arc therapy (VMAT) techniques, in combination with S-1 at a dose of 50 mg twice daily. Target delineation followed a prespecified, study-specific postoperative contouring protocol developed by the coordinating center before trial initiation and distributed to participating centers. Centralized training and ongoing quality oversight were used to improve interinstitutional consistency. Three to 4 weeks after completing RT, patients received 3 additional cycles of SOX chemotherapy at the same dose as in the induction phase. In the SOX arm, patients received 6 cycles of the SOX regimen alone. The SOX regimen consisted of S-1 (30-40 mg/m^2^ twice daily on days 1-14) and oxaliplatin (130 mg/m^2^ on day 1), repeated every 3 weeks. eFigure 1 in Supplement 2 provides an overview of the trial design.

### Patients

**Figure 1.**
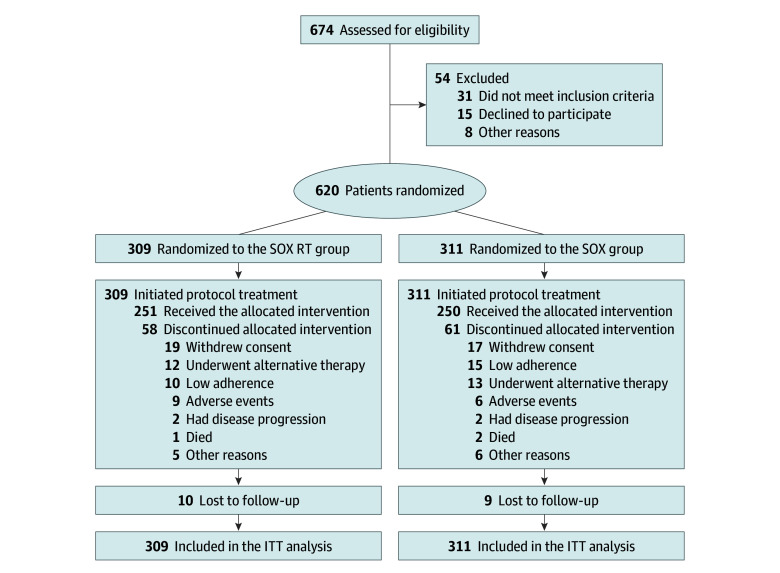
Trial Flow Diagram All randomized patients were included in the intention-to-treat (ITT) analysis. Receipt of the allocated intervention was defined as completion of the prespecified assigned treatment per protocol. SOX indicates S-1 plus oxaliplatin; SOX RT, SOX plus radiotherapy.

The study included adults aged 18 to 70 years with gastric adenocarcinoma who had undergone radical gastrectomy and D2 lymphadenectomy. Eligible patients were required to have a postoperative pathological stage of T4a or higher (T ≥ T4a) or node-positive disease (N > N0), according to the seventh edition (2010) of the American Joint Committee on Cancer (AJCC) and Union for International Cancer Control (UICC) tumor-node-metastasis (TNM) staging criteria. All patients were required to have no metastasis or postoperative recurrence, as confirmed by comprehensive evaluation (M0). Additionally, patients were required to have undergone radical D2 resection with no residual disease (R0). Figure 1 shows the patient flow diagram for the trial.

### End Points and Assessment

The prespecified primary time point for reporting the primary end point was 3 years (3-year DFS). DFS was defined as the time from randomization to the first occurrence of recurrence, a second primary cancer, or death from any cause. Secondary end points included OS and adverse events.

The safety population included all patients who received at least 1 dose of the assigned treatment. During and after treatment, all patients followed a standardized follow-up schedule (at least once every 6 months within the first year after enrollment, then at least once every 6-12 months after the treatment was completed). Symptoms, adverse events (graded according to the Common Terminology Criteria for Adverse Events, version 4.0), and laboratory and imaging findings were documented throughout the follow-up period.

### Sample Size Calculation

We performed an a priori sample size calculation for the 3-year DFS. Assuming a 2-sided α = .05 and 80% power, we hypothesized an absolute improvement in 3-year DFS from 70% in the SOX group to 78% in the SOX RT group. Using a 2-sample comparison of proportions, we estimated that 516 participants would be required. Allowing for up to 20% attrition (eg, loss to follow-up or treatment discontinuation), the target enrollment was 620 participants.

### Statistical Analysis

All clinical data were analyzed from January 14 to March 31, 2025, using IBM SPSS Statistics, version 29.0 (IBM Corp), and R, version 4.2.1 (R Project for Statistical Computing). Statistical analyses were conducted in accordance with the intention-to-treat (ITT) principle.

Categorical variables were compared using the χ^2^ test or Fisher exact test, as appropriate. The Kaplan-Meier method was used to estimate DFS and OS, and the log-rank test was used to compare survival curves. HRs and 95% CIs for the treatment effect on DFS and OS were estimated using unadjusted Cox proportional hazards regression models with treatment group as the only covariate, consistent with the randomized design, in the ITT population.

Censoring was defined separately for DFS and OS. For DFS, patients without recurrence, a second primary cancer, or death were censored at the date of the last disease assessment, at which point they were confirmed to be event free. For OS, patients who were alive were censored at the date of last known contact. Patients lost to follow-up or who withdrew consent were censored according to the same end point–specific rules (at the last disease assessment for DFS; at the last known contact for OS). The median follow-up time was calculated using the reverse Kaplan-Meier method. A 2-sided *P* < .05 was considered to be statistically significant. Subgroup analyses were conducted by fitting unadjusted Cox proportional hazards regression models within each subgroup, and the subgroup dot plots display the corresponding within-subgroup treatment effect estimates.

## Results

Between December 1, 2012, and August 30, 2022, a total of 620 patients were enrolled across 5 centers in China and randomly assigned to receive SOX (n = 311) or SOX RT (n = 309) (Figure 1). Patients had a median (IQR) age of 55 (47-62) years and included 401 males (64.7%) and 219 females (35.3%). Among these patients, 274 (44.2%) had T4 stage disease, 590 (95.2%) had node-positive disease, and 436 (70.3%) had stage III disease (according to the AJCC/UICC staging criteria). In the SOX RT group, 58 patients (18.8%) did not receive the assigned treatment. The planned chemotherapy cycles and RT were completed by 250 patients (80.4%) in the SOX group and 251 patients (81.2%) in the SOX RT group. The primary reason for early discontinuation was withdrawal of consent. Overall, baseline patient and tumor characteristics were well balanced between the 2 arms (Table).

**Table.** Baseline Patient Characteristics

Characteristic	Patients, No. (%)			Standardized difference
	All (N = 620)	SOX RT group (n = 309)	SOX group (n = 311)	
Sex				
Female	219 (35.3)	100 (32.4)	119 (38.3)	0.124
Male	401 (64.7)	209 (67.6)	192 (61.7)	
Age, y				0.173
>60	183 (29.5)	79 (25.6)	104 (33.4)	
≤60	437 (70.5)	230 (74.4)	207 (66.6)	
Tumor site				
Gastric antrum	310 (50.0)	155 (50.2)	155 (49.8)	0.084
Gastric body	209 (33.7)	101 (32.7)	108 (34.7)	
Cardia	90 (14.5)	46 (14.9)	44 (14.1)	
Whole stomach	11 (1.8)	7 (2.3)	4 (1.3)	
Tumor size, cm				
≤5	354 (57.1)	181 (58.6)	173 (55.6)	0.060
>5	266 (42.9)	128 (41.4)	138 (44.4)	
Lauren classification^a^				
Mixed	150 (24.2)	85 (27.5)	65 (20.9)	0.174
Intestinal	168 (27.1)	75 (24.3)	93 (29.9)	
Diffuse	302 (48.7)	149 (48.2)	153 (49.2)	
Positive lymph node ratio, %				
≤25	373 (60.2)	190 (61.5)	183 (58.8)	0.054
>25	247 (39.8)	119 (38.5)	128 (41.2)	
Lymphovascular invasion				
Yes	275 (44.4)	140 (45.3)	135 (43.4)	0.119
No	220 (35.5)	114 (36.9)	106 (34.1)	
Unknown	125 (20.2)	55 (17.8)	70 (22.5)	
Perineural invasion				
Yes	358 (57.7)	191 (61.8)	167 (53.7)	0.173
No	181 (29.2)	84 (27.2)	97 (31.2)	
Unknown	81 (13.1)	34 (11.0)	47 (15.1)	
T stage				
Non-T4	346 (55.8)	166 (53.7)	180 (57.9)	0.084
T4	274 (44.2)	143 (46.3)	131 (42.1)	
N stage				
N1	115 (18.5)	58 (18.8)	57 (18.3)	0.093
N3	283 (45.6)	137 (44.3)	146 (46.9)	
N2	192 (31.0)	101 (32.7)	91 (29.3)	
N0	30 (4.8)	13 (4.2)	17 (5.5)	
AJCC/UICC stage				
I	24 (3.9)	9 (2.9)	15 (4.8)	0.102
II	160 (25.8)	79 (25.6)	81 (26.0)	
III	436 (70.3)	221 (71.5)	215 (69.2)	

Abbreviations: AJCC/UICC, American Joint Committee on Cancer/Union for International Cancer Control tumor-node-metastasis (TNM) staging criteria, seventh edition (2010); SOX, S-1 plus oxaliplatin; SOX RT, SOX plus radiotherapy.

^a^ Lauren classification categorizes gastric adenocarcinoma as intestinal, diffuse, or mixed histologic type. Intestinal type is characterized by gland formation and cohesive tumor cells, diffuse type by poorly cohesive tumor cells, and mixed type by both intestinal and diffuse components.

### Long-Term DFS and OS

**Figure 2.**
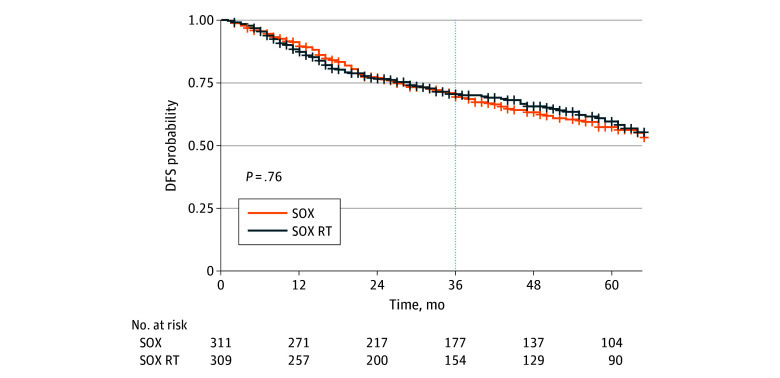
Kaplan-Meier Curves for Disease-Free Survival (DFS) in the Intention-to-Treat Population Patients were randomized to receive S-1 plus oxaliplatin (SOX; n = 311) or SOX plus radiotherapy (SOX RT; n = 309) after D2 gastrectomy. Tick marks indicate censored observations. The dashed vertical line indicates 36 months. *P* value was calculated using a 2-sided log-rank test.

**Figure 3.**
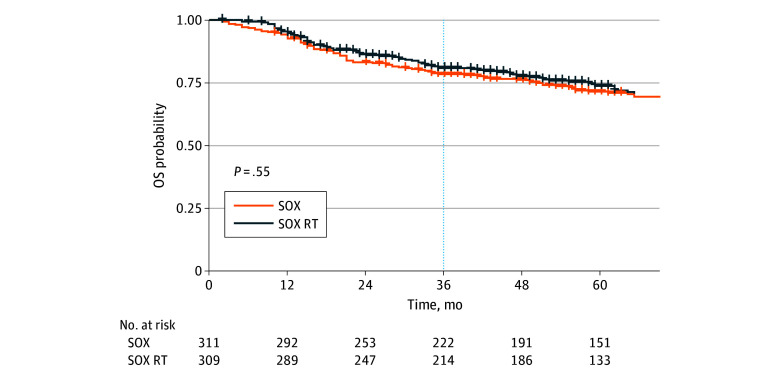
Kaplan-Meier Curves for Overall Survival (OS) in the Intention-to-Treat Population Patients were randomized to receive S-1 plus oxaliplatin (SOX; n = 311) or SOX plus radiotherapy (SOX RT; n = 309) after D2 gastrectomy. Tick marks indicate censored observations. The dashed vertical line indicates 36 months. *P* value was calculated using a 2-sided log-rank test.

The data cutoff for this analysis was November 30, 2024. The median (IQR) follow-up period was 63 (36-92) months, during which 273 DFS events occurred. For the prespecified primary end point of 3-year DFS, there was no evidence of a between-group difference (SOX RT vs SOX: HR, 0.98; 95% CI, 0.73-1.33). Similarly, OS did not differ between groups (HR, 0.86; 95% CI, 0.60-1.23). The Kaplan-Meier–estimated 3-year DFS rates were 70.5% in the SOX RT group and 69.3% in the SOX group (log-rank *P* = .93), and the corresponding 3-year OS rates were 80.8% and 78.4%, respectively (log-rank *P* = .41). At 5 years, the DFS rates were 60.0% and 57.3% in the SOX RT and SOX groups, respectively (log-rank *P* = .76), and the corresponding OS rates were 73.7% and 71.4% (log-rank *P* = .55) (Figure 2 and Figure 3). In a post hoc per-protocol sensitivity analysis (SOX: n = 250; SOX RT: n = 251), results were consistent with the ITT analysis, with no significant between-group differences in DFS (HR, 1.04; 95% CI, 0.77-1.39; log-rank *P* = .80) or OS (HR, 1.13; 95% CI, 0.80-1.61; log-rank *P* = .49) (eFigures 2 and 3 in Supplement 2). We also added a post hoc sensitivity multivariable Cox proportional hazards regression analysis for both DFS and OS. The model included treatment group and clinically relevant prognostic variables. The adjusted results were consistent with the primary ITT analysis (eTable 1 in Supplement 2).

### Subgroup Analysis of DFS and OS

**Figure 4.**
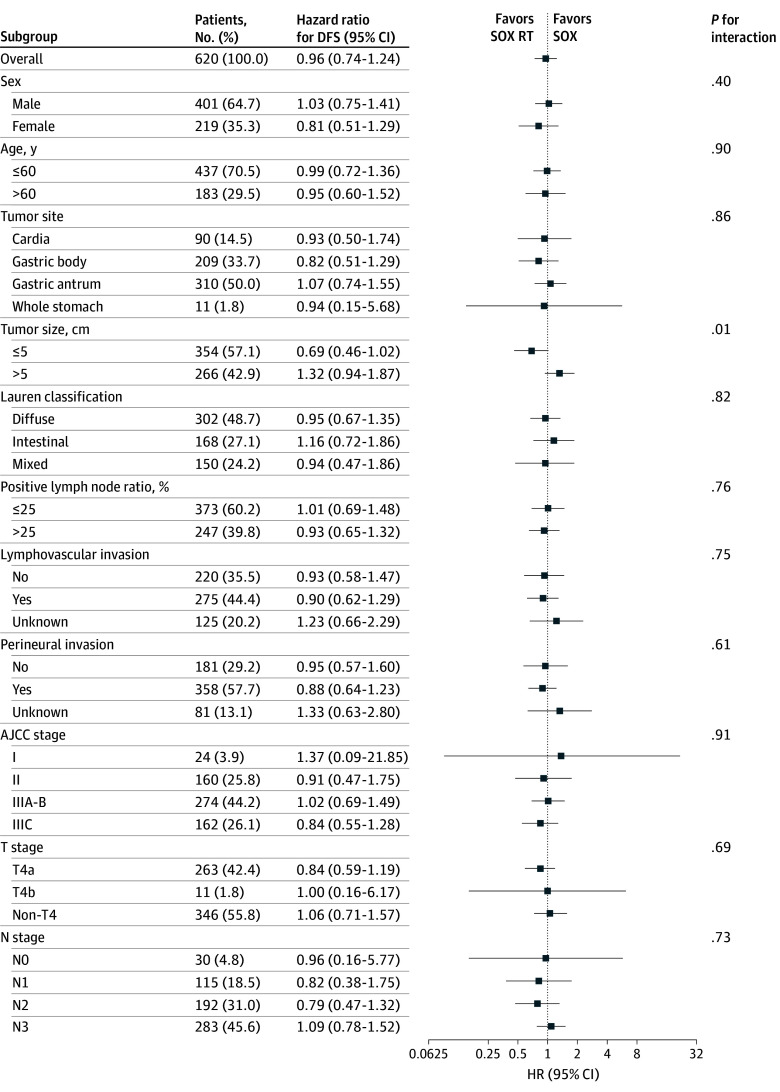
Dot Plot of Subgroup Analyses of Disease-Free Survival (DFS) Hazard ratios (HRs) and 95% CIs were estimated using unadjusted Cox proportional hazards regression models within each subgroup (treatment group as the only covariate). DFS follow-up was censored at 5 years for this subgroup analysis. *P* values for interaction were derived from Cox models that included a treatment-by-subgroup interaction term. HRs less than 1.0 indicate lower risk of disease recurrence or death with SOX plus radiotherapy (SOX RT) compared with S-1 plus oxaliplatin (SOX). Error bars represent 95% CIs. AJCC stage is derived from the seventh edition (2010) of the American Joint Committee on Cancer and Union for International Cancer Control staging criteria.

Subgroup analyses for DFS and OS are shown in Figure 4 and eFigure 4 in Supplement 2. Treatment effects within subgroups were estimated using unadjusted Cox proportional hazards regression models (with treatment group as the only covariate). Overall, the subgroup analyses for DFS did not suggest that the treatment effect differed across baseline subgroups (Figure 4). For OS, the subgroup estimate in patients with T4a disease was an HR of 0.65 (95% CI, 0.43-0.98); however, the interaction test did not indicate heterogeneity of the treatment effect by T stage (*P* for interaction = .11). Accordingly, these subgroup findings are hypothesis-generating and warrant confirmation in future studies (eFigure 4 in Supplement 2).

### Safety

Treatment-related adverse events of any grade occurred in 211 patients (67.8%) in the SOX group and 228 patients (73.8%) in the SOX RT group, with most cases limited to grade 1 or 2 and both groups showing good tolerance. The most common adverse event was leukopenia (104 [33.7%]) in the SOX group, whereas leukopenia (97 [31.2%]) and nausea (97 [31.2%]) were the most frequent adverse events in the SOX RT group. Details are presented in eTable 2 in Supplement 2. No significant differences were observed between the 2 groups.

## Discussion

In recent years, D2 surgery has become the standard treatment in many Asian countries; however, the role of adjuvant chemotherapy combined with RT in the treatment of patients who have undergone radical D2 surgery remains controversial.^16^ To address this concern, we initiated this trial to evaluate the efficacy of 2 adjuvant therapies following D2 gastrectomy. Our results showed that adding RT to the adjuvant SOX regimen did not significantly improve DFS or OS in patients with T4 or node-positive gastric cancer after D2 gastrectomy. Treatment adherence and sensitivity analyses support the robustness of the primary findings.

In the SOX RT group, 58 patients did not complete the planned RT, which may have diluted the treatment difference in the ITT analysis. However, findings from a post hoc per-protocol sensitivity analysis remained consistent with those of the primary analysis, showing no significant between-group differences in DFS or OS. In exploratory subgroup analyses, the OS estimate was an HR of 0.65 and no heterogeneity of the treatment effect by T stage was found. Therefore, the T4a signal should be interpreted as hypothesis-generating rather than definitive evidence for subgroup-specific use, and it requires prospective confirmation.

The finding from the ACTS-GC and CLASSIC trials^8,10,11^ that adjuvant chemotherapy improved survival compared with surgery alone after D2 gastrectomy established systemic therapy as a key postoperative backbone in gastric cancer care. Subsequent intensification strategies have focused on optimizing chemotherapy regimens in higher-risk disease (eg, stage III), as supported by trials such as JACCRO GC-07.^14^ Evidence specifically addressing RT after D2 surgery has been mixed. The ARTIST trial found no overall improvement in 3-year DFS with postoperative CRT after D2 resection, although exploratory subgroups (eg, those with higher nodal burden) suggested possible DFS benefit.^17^ Similarly, the ARTIST 2 trial showed no DFS difference between SOX and SOX RT after D2 resection,^12^ supporting the view that RT is unlikely to add meaningful benefit for the overall node-positive, stage II or III population when effective chemotherapy is delivered.

Conducted contemporaneously with our trial, ARTIST 2 addressed a similar question in patients with D2-resected, node-positive gastric cancer but differed from our trial in several important respects. First, the enrolled populations were not identical. ARTIST 2 included patients with stage II or III, node-positive disease after D2 resection, whereas the present trial focused on a high-risk population defined by T4 and/or node-positive disease. Second, the RT dose and technique differed between studies. ARTIST 2 used 3D-CRT with a dose of 45 Gy, whereas our study predominantly used IMRT or VMAT with a dose of 50.4 Gy. Third, ARTIST 2 was reported after early termination of accrual and with fewer patients than originally planned, whereas this trial has been completed and has a long-term follow-up.

Similarly, a recent randomized clinical trial by Qiao et al^18^ reported no overall benefit of adding postoperative CRT to a SOX-based adjuvant backbone after D2 resection. However, their treatment strategy differed from ours in several important respects, including the sequencing and RT dose (RT was delivered after 4 to 6 cycles of SOX at 45 Gy in 25 fractions with concurrent S-1 vs RT was incorporated earlier in the treatment course and used 50.4 Gy in 28 fractions) as well as the definition of the high-risk subgroup (Qiao et al^18^ classified high-risk disease as pN stage≥N2 plus extraperigastric lymph node metastasis). Altogether, the variability in subgroup signals across these trials likely reflects differences in treatment design and subgroup definitions as well as the limited power and multiplicity inherent in subgroup analyses. Thus, such findings should be interpreted cautiously and viewed as hypothesis-generating pending prospective validation.

Interpretation of postoperative RT trials should be grounded in the surgical context, particularly the extent of lymphadenectomy. Because the Intergroup-0116 and CRITICS trials were conducted largely in settings with less extensive nodal dissection (D0/D1), their findings are not directly transferable to a D2-treated population.^19,20,21^ A plausible explanation is that D2 lymphadenectomy improves baseline locoregional control, reducing the remaining preventable locoregional failure risk and, consequently, the incremental contribution of RT, while systemic recurrence becomes a more important determinant of prognosis and is more directly addressed by effective systemic chemotherapy.

In the safety analysis, adverse events were generally manageable and consistent with expectations. RT was predominantly delivered using contemporary techniques (IMRT or VMAT) to 50.4 Gy in 28 fractions. IMRT or VMAT can improve target conformity and organ-at-risk sparing compared with 3D-CRT,^22^ supporting the applicability of our findings to current practice. Despite its overall consistency with prior trials, our study adds to the literature by enrolling a prespecified high-risk population after R0 D2 resection, using modern RT delivery, and providing long follow-up (median [IQR], 63 [36-92] months) to estimate 5-year outcomes.

In recent years, multimodality strategies have also been explored in the perioperative setting. The TOPGEAR trial did not show survival improvement with adding preoperative CRT to perioperative chemotherapy.^23^ Taken together with our results, further advances may depend more on improving systemic control and biomarker-guided risk stratification than on routine RT intensification for unselected patients with D2 resection. Consistent with this direction, targeted therapies for biomarker-defined subgroups, such as ERBB2 (formerly HER2)-positive disease and claudin18.2-positive advanced gastric cancer, have shown promising activity.^24,25,26^ Future adjuvant trials may therefore prioritize biomarker-guided escalation strategies rather than uniform addition of RT.

### Limitations

This study has several limitations. First, because patients older than 70 years were not enrolled, the findings may not be fully generalizable to older populations. Second, enrollment slowed during the later phase of the trial, extending the overall timeline and delaying completion of the planned analyses, which may limit the timeliness of the results. Third, the trial evaluated a postoperative adjuvant strategy, which may limit applicability in contemporary settings where neoadjuvant treatment is increasingly adopted.

## Conclusions

This trial showed that adding RT to the SOX chemotherapy regimen did not significantly improve DFS or OS following D2 gastrectomy. The primary findings therefore support chemotherapy alone after D2 gastrectomy as an appropriate adjuvant approach for most patients with T4 or node-positive gastric cancer. Any apparent OS difference in the T4a subgroup should be interpreted cautiously as hypothesis-generating and confirmed in future studies.

## Notes

Supplement 1.
Trial Protocol

Supplement 2. **eTable 1.** Multivariable Cox Proportional Hazards Regression Analyses for Disease-Free Survival and Overall Survival **eTable 2.** All Grade and Maximum Grade Adverse Events **eFigure 1.** Trial Design and Treatment Schema **eFigure 2.** Per-Protocol Disease-Free Survival **eFigure 3.** Per-Protocol Overall Survival **eFigure 4.** Subgroup Analyses of Overall Survival

Supplement 3.
Data Sharing Statement
