# Supplementary material for: Adjuvant Chemoradiotherapy or Chemotherapy After D2 Gastrectomy in Gastric Cancer: A Randomized Clinical Trial
Source: JAMA Netw Open. 2026 Jun 15;9(6):e2616154. doi: 10.1001/jamanetworkopen.2026.16154 (PMC13270272; doi:10.1001/jamanetworkopen.2026.16154)
Supplement: Supplement 1. — Trial Protocol [file jamanetwopen-e2616154-s001.pdf]

Protocol

Title: Adjuvant chemoradiotherapy compared with adjuvant chemotherapy for gastric cancer after D2 gastrectomy: a multicenter phase III randomised controlled trial

Version number: Version 1.4
Version date: August 30, 2012
Center: West China Hospital

Principal Investigator:

Xin Wang, MD, Division of Abdominal Tumor Multimodality Treatment, Cancer Center, West China Hospital of Sichuan University, Chengdu, Sichuan Province, China; West China Hospital, Sichuan University, No. 37 Guoxue Alley, Chengdu, 610041, Sichuan Province, China.
e-mail: wangxin@wchscu.edu.cn

Feng Bi, MD, Division of Abdominal Tumor Multimodality Treatment, Cancer Center, West China Hospital of Sichuan University, Chengdu, Sichuan Province, China; West China Hospital, Sichuan University, No. 37 Guoxue Alley, Chengdu, 610041, Sichuan Province, China
e-mail: bifengasco@163.com

TABLE OF CONTENTS

1 TRIAL SYNOPSIS 3
2 Background 5
3 Objectives 5
4 Trial design 5
5 Selection of Patients 7
6 Procedures of the study 8
7 Trial Management 9
8 The risks and benefits 9
9 Adverse Events 9
10 Study assessment 10
11 Study Treatment Controls and Assurance 11
12 Ethical requirements 11
13 Appendix 1: Common Toxicity Criteria for Adverse Events (CTCAE) of the US National Cancer Institute (version 4.0) 12
14 Reference 12

1 TRIAL SYNOPSIS

Title	Adjuvant chemoradiotherapy compared with adjuvant chemotherapy for gastric cancer after D2 gastrectomy: a multicenter phase III randomised controlled trial
Version number	V1.4, August 30, 2012
Principal Investigator	Dr. Xin Wang, MD; Dr. Feng Bi, MD
Study Center	West China Hospital
Study Design	Prospective, Randomized, Multicenter, open-label, phase III trial
Target population	Gastric carcinoma
Number of patients	620 patients
Objectives	Primary Endpoint: Disease-Free Survival (DFS).<br>Secondary Endpoint: Overall Survival (OS) and treatment-related adverse event.
Inclusion Criteria	(1) Age 18-70 years;<br>(2) Pathologically confirmed gastric cancer patients who have undergone surgical resection;<br>(3) Surgery is radical, achieving R0 resection, and D2 lymph node dissection (with organ preservation) (the number of lymph node dissection should be ⩾15);<br>(4) The time to surgery is not more than 3 months;<br>(5) According to the 2010 edition of UICC/AJCC staging criteria, postoperative pathological stage T stage ⩾ T4a, N stage ⩾ N0; and no tumour metastasis or postoperative recurrence is detected by comprehensive examination, i.e., the M stage is M0;<br>(6) Eastern Cooperative Oncology Group (ECOG) performance score: 0-1;<br>(7) Patients who have not received radiotherapy and systemic chemotherapy before or after surgery;<br>(8) Normal function of major organs.<br>(9) Women of childbearing potential must have had a negative pregnancy test (serum or urine) 7 days prior to enrolment and be willing to use an appropriate method of contraception for the duration of the trial and for 8 weeks after the last dose. For males, surgical sterilisation should be applied or agreement to use an appropriate method of contraception for the duration of the trial or 8 weeks after the final dose.<br>(10) Subjects voluntarily enrolled in the study, signed an informed

	consent form, were compliant, and cooperated with follow-up visits.
Exclusion Criteria	(1) Previous or concurrent other malignancies, except cured basal cell carcinoma of the skin and carcinoma in situ of the cervix;<br>(2) Pregnant or breastfeeding women;<br>(3) Patients with severe cardiovascular disease and diabetes that cannot be easily controlled;<br>(4) People with mental disorders;<br>(5) People with serious infections;<br>(6) Hypersensitivity to S-1 or platinum oxalate;<br>(7) Serious gastrointestinal diseases that affect the absorption of oral chemotherapeutic drugs<br>(8) Participation in another clinical trial within 4 weeks prior to the start of treatment.
Randomization	Patients were assigned in a 1:1 ratio to SOXRT arm or SOX arm by block randomization.
Treatment	This is a multicenter, phase III clinical trial led by West China Hospital, Sichuan University. Patients will be randomly assigned to either the SOXRT arm (experimental group) or the SOX arm (control group).<br>SOXRT arm: Patients will receive one cycle of induction chemotherapy with the SOX regimen 21 days prior to radiotherapy initiation, followed by S-1-based concurrent chemoradiotherapy. After completing radiotherapy, patients will undergo three additional cycles of SOX chemotherapy, with the same dosing regimen as in the induction chemotherapy.<br>SOX arm: Patients will receive a total of six cycles of SOX chemotherapy.
Statistical Analysis	Categorical variables were compared with the use of Chi-squared test or Fisher's exact test. Kaplan–Meier method was used to analyses the DFS and OS, log-rank test to assess the significance, and Cox proportional hazards model to calculate hazard ratio (HR). The median follow-up time was calculated by reverse-KM method. Statistical significance was defined as a two-side P value < 0.05.

2 Background

Surgery is the primary modality for the treatment of resectable gastric cancer, and radiotherapy can improve local control rates, but does the benefit of local control with postoperative radiotherapy translate into a survival benefit? The landmark INT-0116 study demonstrated that postoperative adjuvant radiotherapy (45 Gy/25 doses) given after radical surgery for gastric cancer increased 3-year survival from 41% to 50% ($P < 0.001$), and the NCCN guidelines recommend postoperative adjuvant radiotherapy for patients with postoperative stage T2N0M0 and above. However, the lack of standardisation of the surgical approach and the fact that D2 resection accounted for only 10% of the patients in this study have led to ongoing controversy. To address this issue, the Korean ARTIST phase III study focused on postoperative D2 patients and showed that concurrent radiotherapy (45 Gy/25 sessions) improved DFS by 5.2% in the lymph node-positive subgroup, although it did not improve overall DFS ($P=0.0365$). This result suggests the need to re-evaluate the precise benefit of radiotherapy in the post-D2 population.

Moreover, our department has previously conducted a phase I/II clinical study of the FOLFOX regimen combined with synchronous radiotherapy (at a dose of 50.4 Gy) after gastric cancer surgery, which confirmed the efficacy and safety of 50.4 Gy of radiotherapy combined with synchronous chemotherapy.The results of the phase II clinical study showed that the 1- and 2-year OS and DFS of the FOLFOX regimen combined with synchronous after radical D2 surgery were 95%, 82% and 85%, 78%. And the phase I/II clinical study of SOX regimen combined with synchronous radiotherapy after gastric cancer has been completed, after which the maximum tolerated dose (maximum tolerated dose, MDT) and safety of S-1 during synchronous radiotherapy have been explored. On this basis, I expect to further develop this multicenter phase III randomized controlled clinical study to explore the value of SOX regimen combined with simultaneous radiotherapy in the adjuvant treatment of gastric cancer patients after D2 radical surgery.

3 Objectives

The patients after D2 radical surgery for gastric cancer that meet the enrolment criteria were randomly assigned to be given SOX regimen combined with simultaneous radiotherapy or chemotherapy with SOX regimen alone, to explore the value of simultaneous radiotherapy after D2 radical surgery for gastric cancer.
(1) The primary objective was: disease free survival (DFS).
(2) The secondary objectives were: overall survival (OS), and various types of adverse events.

4 Trial design

4.1 Randomisation: patients who met the inclusion criteria and signed the informed consent

were randomly grouped into a synchronous radiotherapy group (experimental group) and a chemotherapy-only group (control group) in a 1:1 ratio.

4.2 The protocol of the experimental group: one cycle of induction chemotherapy (SOX) was administered 21 days before radiotherapy; the specific dosage of SOX was S-1 30-40mg/m2 bid D1-14, oxaliplatin 130mg/m2 D1,Q3W, and then S-1 simultaneous radiotherapy was started, and the dosage of S-1 was 50mg bid during the period of simultaneous radiotherapy, which was used in combination with radiotherapy for 5 days a week for a consecutive period. in combination, 5 days per week for 28 days. After 3-4 weeks of radiotherapy, the SOX regimen was administered for 3 cycles at the same dose as the induction chemotherapy.

4.3 Control regimen: A total of 6 cycles of SOX regimen (at the same dose as in the experimental group) will be given.

4.4 Radiotherapy: radiotherapy was started within 3 months after surgery. The radiotherapy dose was 50.4 Gy/28f, 1.8 Gy/d, 5f/w. Radiotherapy was given on the 1st-5th day of each week for 28 consecutive days; radiotherapy was given by three-dimensional conformal radiotherapy (3D-CRT) or intensity-modulated radiotherapy (IMRT).

i. Radiotherapy localisation: fasting for 4 hours before localisation; supine position, hands on elbows, body mould fixation, CT simulation localisation, enhanced scanning, scanning range from T6 lower edge to L4 lower edge, layer thickness 3mm.

ii. Radiotherapy target zone (CTV) reference: postoperative radiotherapy target zone of gastric cancer includes tumour bed, anastomosis and regional lymph nodes. For the specific outlining method of target area, refer to 'Annex 1 Specification for Outlining Target Area of Postoperative Radiotherapy for Gastric Cancer' provided in this protocol.

iii. Radiotherapy plan: 3D-CRT or IMRT technology is used; PTV is externally applied in the anterior-posterior and left-right directions by 0.8 cm on the basis of CTV, and externally applied in the up-and-down direction by 1.0 cm; 95% of the PTV has a minimum dose of 50.4 Gy/28f; hotspot dose is ⩽ 107% of the prescribed dose, and the volume is ⩽ 2 CC; the target area conformity is ⩾ 0.8; spinal cord is ⩽ 40 Gy, and the liver V30 is ⩽ 33% of the dose. The maximum dose received by 33% of the volume of one kidney (mostly right kidney) is <15 Gy, and the average of both kidneys is <15 Gy; D50 of small intestine is <20-30 Gy.

4.5 Effectiveness evaluation: patients should undergo effectiveness evaluation at least once every 6 months during the first year after enrolment; after the end of treatment, regular follow-up and effectiveness evaluation should be conducted in accordance with the requirements of the supervising physician, and effectiveness evaluation should be conducted

at least once every 6-12 months. The time intervals of the evaluation should all be referenced to the point in time when the patient was randomised to the group. The evaluation includes physical examination, imaging (CT, chest radiograph, ultrasound, etc.) and laboratory tests (routine blood, blood biochemistry, tumour markers, etc.). The primary study endpoint was 3-year DFS, which was defined as the time from randomisation to the occurrence of tumour recurrence/metastasis (i.e., disease progression), or death in the randomised group; the secondary study endpoint of 3-year OS referred to the time from randomisation to death; and safety was also included.

5 Selection of Patients

5.1 Inclusion Criteria

(1) Age 18-70 years;

(2) Gastric cancer patients who have been diagnosed by pathology and have been surgically resected;

(3) Surgery is radical, achieving R0 resection, and D2 lymph node dissection (with organ preservation) (the number of lymph node dissection should be ⩾15);

(4) The time to surgery is not more than 3 months;

(5) According to the 2010 edition of UICC/AJCC staging criteria, postoperative pathological stage T stage ⩾ T4a, N stage ⩾ N0; and no tumour metastasis or postoperative recurrence is detected by comprehensive examination, i.e., the M stage is M0;

(6) ECOG PS score: 0-1;

(7) Patients who have not received radiotherapy and systemic chemotherapy before or after surgery;

(8) Normal function of major organs, i.e., the following indications are met:

i. routine blood tests need to meet the following criteria: HB ⩾9g/dL, WBC ⩾3.5/4.0×109/L, PLT⩾ 100×109/L

ii. Biochemical tests must meet the following criteria: Crea and BIL ⩽ 1.0 times the upper limit of normal (ULN), ALT and AST ⩽ 2.5 times the upper limit of normal (ULN).

(9) Women of childbearing potential must have had a negative pregnancy test (serum or urine) 7 days prior to enrolment and be willing to use an appropriate method of contraception for the duration of the trial and for 8 weeks after the last dose. For males, surgical sterilisation should be applied or agreement to use an appropriate method of contraception for the duration of the trial or 8 weeks after the final dose.

(10) Subjects voluntarily enrolled in the study, signed an informed consent form, were compliant, and cooperated with follow- up.

5.2 Exclusion Criteria

(1) Previous or concurrent other malignancies, except cured basal cell carcinoma of the skin and carcinoma in situ of the cervix;

(2) Pregnant or breastfeeding women;

(3) Patients with severe cardiovascular disease and diabetes that cannot be easily controlled;

(4) People with mental disorders;

(5) People with serious infections;

(6) Hypersensitivity to S-1 or platinum oxalate;

(7) Serious gastrointestinal diseases that affect the absorption of oral chemotherapeutic drugs

(8) Participation in another clinical trial within 4 weeks prior to the start of treatment.

5.3 Reduction and discontinuation of treatment

5.3.1 Patients can voluntarily withdraw from the study at any time.

5.3.2 In the event of a 3 or 4 grade haematological toxicity, the next cycle of chemotherapy needs to be given after the toxicity has returned to 0-1 grade and the dose of the next cycle of chemotherapy, S-1, is reduced by 25%.

5.3.3 In the event of ⩾2 grade of non-haematological toxicity, chemotherapy should be delayed by 1 week. In the event of 4 grade toxicity during concurrent radiotherapy, other than alopecia, chemotherapy is discontinued and radiotherapy is continued whenever it is considered to be chemotherapy-related.

5.3.4 Discontinue radiotherapy only if radiotherapy-associated 3 grade skin inflammation occurs, until toxicity returns to 0-1 grade. If the patient's recovery period requires more than 3 weeks, treatment should be discontinued and the patient discharged from the group.

5.3.5 Toxicity was graded according to CTCAE 4.0 criteria.

6 Procedures of the study

(1) Patients meeting the enrolment criteria were included in this study after signing the informed consent form.

(2) Patients were treated in strict accordance with the methods of this study.

(3) Observe and follow up the patients during and after the treatment according to the methods of side effects and efficacy evaluation of this study.

(4) To write a paper based on the data obtained from the study.

7 Trial Management

The informed consent process involves a thorough explanation of the trial's objectives, procedures, and potential risks/benefits by the principal investigator. Participants must sign an Informed Consent Form (ICF), which includes both informational and signature pages. This process must comply with ethical standards, including the Declaration of Helsinki and CFDA's Good Clinical Practice (GCP) guidelines. Any protocol modifications require prior approval from the ethics committee. The original ICF is securely archived at the research site, while participants receive a copy for their records.

8 The risks and benefits

8.1 The safety profile of this study is excellent and the risk to enrolled patients is low. The main side effects and adverse reactions that may occur during the study include: nausea, vomiting, fatigue, nausea, diarrhoea, abdominal pain, and leukocyte/platelet reduction. Phase I/II clinical studies have confirmed that the SOX regimen combined with simultaneous radiotherapy has a low incidence of both 3rd/4th degree toxicities and side effects, with nausea and leukopenia being the highest incidence; no life-threatening serious adverse effects occurred.

8.2 The benefit of this study is that it can explore the value of the SOX regimen combined with simultaneous radiotherapy after D2 radical surgery for gastric cancer, as well as its efficacy and safety, and find a more effective and safer treatment option for postoperative gastric cancer patients.

9 Adverse Events

9.1 Definition of AE

An Adverse Event (AE) refers to any undesirable medical occurrence in a study participant that coincides with the administration of an investigational drug, regardless of its causal relationship. This encompasses any unfavorable or unintended symptoms, signs, or medical conditions, whether treatment-related or not, including laboratory abnormalities necessitating clinical intervention (e.g., dose adjustment or treatment discontinuation). AEs are recorded from the moment informed consent is obtained until the completion of the protocol-defined follow-up period.

A Serious Adverse Event (SAE) is an AE that meets at least one of the following criteria: results in death, poses an immediate life-threatening risk, necessitates hospitalization or extends an existing hospital stay, leads to significant or persistent

disability/incapacity, involves a congenital anomaly or birth defect, or is deemed a medically critical event requiring intervention to prevent these outcomes. All SAEs must be reported within 24 hours of awareness, irrespective of causality. Investigators are responsible for assessing the potential relationship between the intervention and the event, ensuring thorough documentation and follow-up until resolution or stabilization.

9.2 Assessment of AE

All toxicity evaluations are evaluated based on the Common Terminology Criteria for Adverse Events (CTCAE) v4.0, published by the U.S. National Cancer Institute (NCI). The CTCAE v4.0 provides standardized grading criteria for AEs, categorizing their severity from Grade 1 to Grade 5, defined as follows: Grade 1 = mild, Grade 2 = moderate, Grade 3 = severe, Grade 4 = life-threatening, and Grade 5 = death. Radiotherapy-related adverse events are assessed using the Radiation Therapy Oncology Group (RTOG) Acute Radiation Morbidity Scoring Criteria from the initiation of treatment until 28 days post-radiation. Evaluations are conducted weekly during therapy and biweekly during follow-up, incorporating clinician-reported outcomes and patient-reported symptom diaries. The primary assessment domains include: dermatologic toxicity, mucosal injury, gastrointestinal toxicity, pulmonary complications.

10 Study assessment

10.1 Effective evaluation indicators: The primary purpose is: DFS. The secondary purpose is: and OS.

10.2 Safety evaluation index: including all toxic side effects and adverse events observed during the trial.

10.3 Sample content estimation: we hypothesised that synchronous radiotherapy would result in higher DFS. with a double significance level of 0.05 and a certainty of 80%. We expect that the 3-year DFS of SOX regimen combined with simultaneous radiotherapy can be at least 8% higher than that of the chemotherapy-only group, i.e., from 70% to 78%. Based on the sample size formula for the comparison of the two sample rates, the sample size needed for this project was calculated to be 516 patients; assuming that 20% of the patients would need to be excluded from the statistics, the total number of patients that would need to be enrolled in this project would be 620.

10.4 Statistical methods

All clinical data were analyzed using IBM SPSS Statistics (IBM Corp., Armonk, NY, USA. version 29.0) and R software (version 4.2.1). Categorical variables were compared with the use of Chi-squared test or Fisher's exact test. Kaplan–Meier method was used to analyses the DFS and OS, log-rank test to assess the significance, and Cox

proportional hazards model to calculate hazard ratio (HR). The median follow-up time was calculated by reverse-KM method. Statistical significance was defined as a two-side P value < 0.05.

11 Study Treatment Controls and Assurance

11.1 Quality control measures:

(1) All investigators should enrol and treat patients in strict accordance with the trial plan.

(2) The grading of all toxic reactions should be judged according to the grading criteria specified in the trial.

(3) The outlining of the radiotherapy target area, and the preparation requirements before radiotherapy, etc. should be carried out in strict accordance with the provisions of Oncology Radiotherapy.

(4) Inform the subjects of the possible adverse reactions during the trial, and the treatment methods to be adopted in case of adverse reactions.

11.2 Quality assurance measures:

(1) The person in charge of the study strengthens the quality supervision of this trial.

(2) The radiotherapy target area of patients should be carried out in strict accordance with the target area outlining specifications of this study to ensure the consistency of the radiotherapy target area.

(3) The person in charge of the study is responsible for supervising and ensuring that the rights and interests of the subjects in the clinical trial are safeguarded, that the trial records and reported data are accurate, complete and error-free, and that the trial follows the approved study protocol and relevant regulations.

12 Ethical requirements

This clinical trial will be conducted in accordance with the Helsinki (2002 version) and China's relevant clinical trial research norms and regulations. The trial protocol will be approved and ratified by the Ethics Committee of West China Hospital of Sichuan University before the start of the trial. Before each patient is enrolled in the study, it is the responsibility of the investigating physician to provide him/her or his/her designated representative with a complete and comprehensive description of the purpose, procedures, and possible risks of the study in written text. Patients should be made aware of their right to withdraw from this study at any time. A written Patient Informed Consent form (included as an appendix to the protocol) shall be given to each patient prior to enrolment and it is the responsibility of the investigating physician to obtain informed consent prior to enrolment of each patient in the

study.

13 Appendix 1: Common Toxicity Criteria for Adverse Events (CTCAE) of the US National Cancer Institute (version 4.0)

The Common Terminology Criteria for Adverse Events (CTCAE) Version 4.0, developed by the U.S. National Cancer Institute (NCI), provides a standardized framework for assessing and grading the severity of adverse events (AEs) in oncology clinical trials and patient management. The complete CTCAE v4.0 document is available at:https://ctep.cancer.gov/protocolDevelopment/electronic_applications/ctc.htm#ctc_archive

14 Reference

1. Xin Wang, Yongsheng Wang, Meng Qiu, et al. Postoperative chemoradiotherapy in gastric cancer: a phase I study of radiotherapy with dose escalation of oxaliplatin, 5-fluorouracil, and leucovorin (FOLFOX regimen). Med Oncol. 2011 Dec;28 Suppl 1:S274-9. Epub 2010 Nov 30.
2. Lee J,Lim do H,Kim S, et al. Phase III Trial Comparing Capecitabine Plus Cisplatin Versus Capecitabine Plus Cisplatin With Concurrent Capecitabine Radiotherapy in Completely Resected Gastric Cancer With D2 Lymph Node Dissection: The ARTIST Trial. J Clin Oncol. 2012 Jan 20;30(3):268-73. Epub 2011 Dec 19.
3. Bang YJ,Kim YW,Yang HK, et al. Adjuvant capecitabine and oxaliplatin for gastric cancer after D2 gastrectomy (CLASSIC): a phase 3 open-label, randomised controlled trial. Lancet.2012 Jan 28;379(9813):315-21. Epub 2012 Jan 7.
4. Macdonald JS,Smalley SR,Benedetti J, et al. Chemoradiotherapy after surgery compared with surgery alone for adenocarcinoma of the stomach or gastroesophageal junction. N Engl J Med. 2001 Sep 6;345(10):725-30.
5. Sakuramoto S,Sasako M,Yamaguchi T, et al. Adjuvant Chemotherapy for Gastric Cancer with S-1, an Oral Fluoropyrimidine. N Engl J Med. 2007 Nov 1;357(18):1810-20.
6. Smalley SR, Benedetti JK, Haller DG, et al. Updated Analysis of SWOG-Directed Intergroup Study 0116:A Phase III Trial of Adjuvant Radiochemotherapy Versus Observation After Curative Gastric Cancer Resection. J Clin Oncol. 2012 Jul 1;30(19):2327-33.
7. Meyerhardt JA, Fuchs CS. Adjuvant therapy in gastric cancer: can we prevent recurrences? Oncology 2003; 17: 714–722.
8. Di Costanzo F, Gasperoni S, Manzione L， et al. Adjuvant chemotherapy in completely resected gastric cancer: a randomized phase III trial conducted by GOIRC. J Natl Cancer Inst. 2008 Mar 19;100(6):388-98. Epub 2008 Mar 11.
9. De Vita F, Giuliani F, Orditura M, et al. Adjuvant chemotherapy with epirubicin,leucovorin, 5-fluorouracil and etoposide regimen in resected gastric cancer patients: a randomized phase III trial

by the Gruppo Oncologico Italia Meridionale (GOIM 9602 Study). Ann Oncol. 2007 Aug;18(8):1354-8. Epub 2007 May 24.

10. Nitti D, Wils J, Dos Santos JG, et al. Randomized phase III trials of adjuvant FAMTX or FEMTX compared with surgery alone in resected gastric cancer. A combined analysis of the EORTC GI Group and the ICCG. Ann Oncol. 2006 Feb;17(2):262-9. Epub 2005 Nov 17.
11. Hallissey MT, Dunn JA, Ward LC et al.The second British Stomach Cancer Group trial of adjuvant radiotherapy or chemotherapy in respectable gastric cancer: five-year follow-up. Lancet. 1994; 343: 1309-1312.
12. Moerte CG, Childs DS, O'Fallon JR et al. Combined 5-fluorouracil and radiation therapy as a surgical adjuvant for poor prognosis gastric carcinoma. J Clin Oncol 1984; 2: 1249–1254.
13. Atiq OT, Kelsen DP, Shiu MH, et al, Phase II trial of postoperative adjuvant intraperitoneal cisplatin and fluorouracil and systemic fluorouracil chemotherapy in patients with resected gastric cancer. J Clin Oncol. 1993 Mar;11(3):425-33.
14. Jansen EP, Boot H, Dubbelman R, et al. Postoperative chemoradiotherapy in gastric cancer -- a Phase I/II dose-finding study of radiotherapy with dose escalation of cisplatin and capecitabine chemotherapy. Br J Cancer. 2007 Sep 17;97(6):712-6.
15. Jansen EP, Boot H, Saunders MP, et al. A phase I-II study of postoperative capecitabine-based chemoradiotherapy in gastric cancer. Int J Radiat Oncol Biol Phys. 2007 Dec 1;69(5):1424-8. Epub 2007 Aug 6.
16. Beşe NS, Büyükünal E, Ozgüroğlu M,, et al. Toxicity and survival results of a phase II study investigating the role of postoperative chemo-radioimmunotherapy for gastric adenocarcinoma. Strahlenther Onkol. 2005 Oct;181(10):652-9.
17. Chang JS, Lim JS, Noh SH, et al. Patterns of regional recurrence after curative D2 resection for stage III (N3) gastric cancer: Implications for postoperative radiotherapy. Radiother Oncol. 2012 Sep;104(3):367-73.

Appendix 2

Contour Delineation Specifications for Postoperative Radiotherapy Target Volumes in Gastric Cancer

The delineation of postoperative radiotherapy target volumes after D2 radical gastrectomy for gastric cancer primarily refers to the delineation of the CTV (Clinical Target Volume). Substantial differences may exist in target volume delineation across institutions and among clinicians. This may affect the quality of multicenter clinical studies. Therefore, this contouring specification is formulated to improve the consistency of radiotherapy planning in this clinical study.

I. Radiotherapy Simulation

Contrast-enhanced CT simulation is used. Patients should fast for 4 hours before simulation; the position should be supine, with both arms elevated (hands holding the elbows), immobilized with a body mold; the scanning range should extend from the inferior border of T6 to the inferior border of L4, with a slice thickness of 3 mm.

II. CTV Delineation

1. Postoperative radiotherapy target volumes for gastric cancer include the tumor bed, anastomosis, and regional lymph nodes. On each slice of the simulation CT images, the regions requiring radiotherapy should be delineated within a single CTV; that is, each region should not be contoured separately.

(1) Anastomosis:

① For distal one-third gastric cancer, the duodenal stump should be included in the CTV;

② For proximal one-third gastric cancer or tumors at the esophagogastric junction, the esophagojejunostomy should be included in the CTV;

③ For tumors at the esophagogastric junction, the CTV should extend cranially to include 4 cm of the esophagus (to cover paraesophageal lymph nodes).

(2) Tumor bed

① For gastric cancer with T stage T1-2, the CTV does not need to include the tumor bed;
② Anterior abdominal wall: only in gastric cancer with T stage T3-4, when preoperative imaging and/or the intraoperative surgeon's description indicates suspected or confirmed invasion of the anterior abdominal wall, or an indistinct interface between the tumor and the anterior abdominal wall, should the postoperative radiotherapy CTV include the anterior abdominal wall.
(3) Lymphatic drainage regions
The regional lymph nodes to be included in the CTV vary according to the anatomic location of the gastric cancer (proximal one-third, middle one-third, and distal one-third stomach).
① Esophagogastric junction cancer, cardia cancer, or upper one-third gastric cancer: paraesophageal lymph nodes, paracardial lymph nodes (stations 1-2), lymph nodes along the lesser and greater curvatures (stations 3-4), lymph nodes along the left gastric artery (station 7), lymph nodes along the celiac artery (station 9), and lymph nodes along the splenic artery / splenic hilar region (stations 10-11). (Stations 1-4, 7, 9-11)
② Middle gastric cancer (gastric body cancer): paracardial lymph nodes (stations 1-2), lymph nodes along the lesser and greater curvatures (stations 3-6), lymph nodes along the left gastric artery (station 7), lymph nodes along the common hepatic artery (station 8), lymph nodes along the celiac artery (station 9), lymph nodes along the splenic artery / splenic hilar region (stations 10-11), hepatoduodenal ligament lymph nodes (station 12), and lymph nodes in the posterior pancreaticoduodenal region (station 13). (Stations 1-13)
③ Gastric antrum / distal one-third gastric cancer: lymph nodes along the lesser and greater curvatures (stations 3-6), lymph nodes along the left gastric artery (station 7), lymph nodes along the common hepatic artery (station 8), lymph nodes along the celiac artery (station 9), lymph nodes along the splenic artery (station 11), hepatoduodenal ligament lymph nodes (station 12), and lymph nodes in the posterior pancreaticoduodenal region (station 13). (Stations 3-9, 11-13)

Note: When the extent of primary tumor invasion involves more than one anatomic region, the radiotherapy CTV should also include the corresponding lymphatic drainage regions.

III. Radiotherapy Planning

1. 3D-CRT, IMRT should be used;

2. Based on the CTV, the PTV should be expanded by 0.8 cm in the anterior, posterior, left, and right directions, and by 1.0 cm in the cranial-caudal direction;

3. Minimum dose covering 95% of the PTV: 50.4 Gy in 28 fractions; hotspot dose ≤ 107% of the prescription dose, with hotspot volume ≤ 2 cc;

4. Conformity index (CI) ≥ 0.8;

5. Dose constraints for major organs at risk: spinal cord ≤ 40 Gy, liver V30 ≤ 33%, the dose to 33% of the volume of one kidney (most commonly the right kidney) <15 Gy, mean dose to both kidneys <15 Gy; small bowel D50 <20-30 Gy.
