# Supplementary material for: Adjuvant Chemoradiotherapy or Chemotherapy After D2 Gastrectomy in Gastric Cancer: A Randomized Clinical Trial
Source: JAMA Netw Open. 2026 Jun 15;9(6):e2616154. doi: 10.1001/jamanetworkopen.2026.16154 (PMC13270272; doi:10.1001/jamanetworkopen.2026.16154)
Supplement: Supplement 2. — eTable 1. Multivariable Cox Proportional Hazards Regression Analyses for Disease-Free Survival and Overall Survival eTable 2. All Grade and Maximum Grade Adverse Events eFigure 1. Trial Design and Treatment Schema eFigure 2. Per-Protocol Disease-Free Survival eFigure 3. Per-Protocol Overall Survival eFigure 4. Subgroup Analyses of Overall Survival [file jamanetwopen-e2616154-s002.pdf]

Supplemental Online Content

Wang X, Yan O, Zhou J, et al. Adjuvant chemoradiotherapy or chemotherapy after D2 gastrectomy in gastric cancer: a randomized clinical trial. *JAMA Netw Open*. 2026;9(6):e2616154. doi:10.1001/jamanetworkopen.2026.16154

eTable 1. Multivariable Cox Proportional Hazards Regression Analyses for Disease-Free Survival and Overall Survival

eTable 2. All Grade and Maximum Grade Adverse Events

eFigure 1. Trial Design and Treatment Schema

eFigure 2. Per-Protocol Disease-Free Survival

eFigure 3. Per-Protocol Overall Survival

eFigure 4. Subgroup Analyses of Overall Survival

This supplemental material has been provided by the authors to give readers additional information about their work.

© 2026 Wang X et al. *JAMA Network Open*

eTable 1. Multivariable Cox Proportional Hazards Regression Analyses for Disease-Free Survival and Overall Survival

Parameter	Disease-free survival		Overall survival	
	HR (95% CI)	P value	HR (95% CI)	P value
**Age**				
≤ 60	1 [Reference]		1 [Reference]	
> 60	1.28 (0.98-1.67)	.068	1.32 (0.97-1.80)	.075
**Treatment**				
SOXRT	1 [Reference]		1 [Reference]	
SOX	1.08 (0.85-1.37)	.546	1.05 (0.79-1.40)	.723
**T stage**				
T4a	1 [Reference]		1 [Reference]	
T4b	1.00 (0.40-2.47)	.994	1.18 (0.43-3.26)	.751
Non-T4	0.55 (0.35-0.87)	.010	0.56 (0.31-0.99)	.047
**N stage**				
N0	1 [Reference]		1 [Reference]	
N1	1.68 (0.74-3.82)	.215	2.42 (0.69-8.47)	.166
N2	2.11 (0.90-4.92)	.085	2.15 (0.59-7.83)	.247
N3	2.97 (1.11-7.94)	.030	4.13 (0.97-17.64)	.055
**AJCC stage**				
I	1 [Reference]		1 [Reference]	
II	1.92 (0.58-6.34)	.284	1.07 (0.31-3.70)	.913
IIIA-B	1.61 (0.45-5.73)	.460	1.53 (0.40-5.81)	.536
IIIC	1.17 (0.27-5.09)	.837	0.96 (0.18-5.03)	.966
**Positive lymph node ratio**				
≤ 25%	1 [Reference]		1 [Reference]	
> 25%	1.49 (1.08-2.05)	.015	1.51 (1.04-2.21)	.031
**Tumor size**				
≤ 5 cm	1 [Reference]		1 [Reference]	
> 5cm	1.45 (1.13-1.87)	.004	1.46 (1.08-1.97)	.013

Abbreviations: AJCC, American Joint Committee on Cancer; CI, confidence interval; HR, hazard ratio. Reference categories: age ≤ 60 years; treatment group, SOXRT;

© 2026 Wang X et al. *JAMA Network Open*

T stage, T4a; N stage, N0; AJCC stage, I; lymph node positive rate ≤ 25%; tumor size ≤ 5 cm. P values are 2-sided and are shown for comparisons with the reference category.

© 2026 Wang X et al. *JAMA Network Open*

eTable 2. All grade and maximum grade adverse events.

	SOXRT (n=309)		SOX (n=311)		
	ALL grade	Grade 3 or 4	ALL grade	Grade 3 or 4	P
Leucopenia	104（33.7%）	20（6.5%）	97（31.2%）	21（6.8%）	0.568
Neutropenia	85（27.5%）	10（3.2%）	62（19.9%）	4（1.3%）	0.870
Thrombocytopenia	44（14.2%）	3（0.9%）	46（14.8%）	9（2.9%）	0.935
Anaemia	79（25.6%）	18（5.8%）	88（28.3%）	20（6.4%）	0.499
Nauseous	85（27.5%）	10（3.2%）	97（31.2%）	5（1.6%）	0.358
Vomiting	49（15.9%）	3（1.0%）	54（17.3%）	5（1.6%）	0.692
Diarrhea	13（4.2%）	1（0.3%）	10（3.2%）	0（0.0%）	0.659
Constipation	27（8.7%）	3（1.0%）	37（11.9%）	4（1.3%）	0.245
Anorexia	79（25.6%）	18（5.2%）	85（27.7%）	4（1.3%）	0.212
Fatigue	59（19.1%）	0（0.0%）	58（18.6%）	0（%）	0.969

© 2026 Wang X et al. *JAMA Network Open*

eFigure 1. Trial design and treatment schema

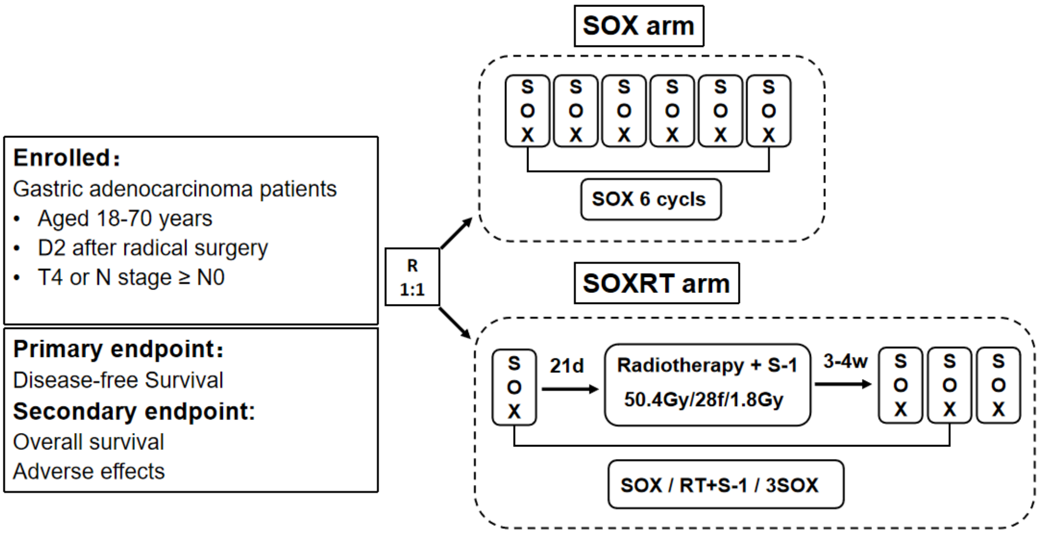

Abbreviations: S-1, chemotherapy; SOX, S-1 plus oxaliplatin; SOXRT, SOX plus chemoradiotherapy.

eFigure 2. Per-Protocol Disease-Free Survival

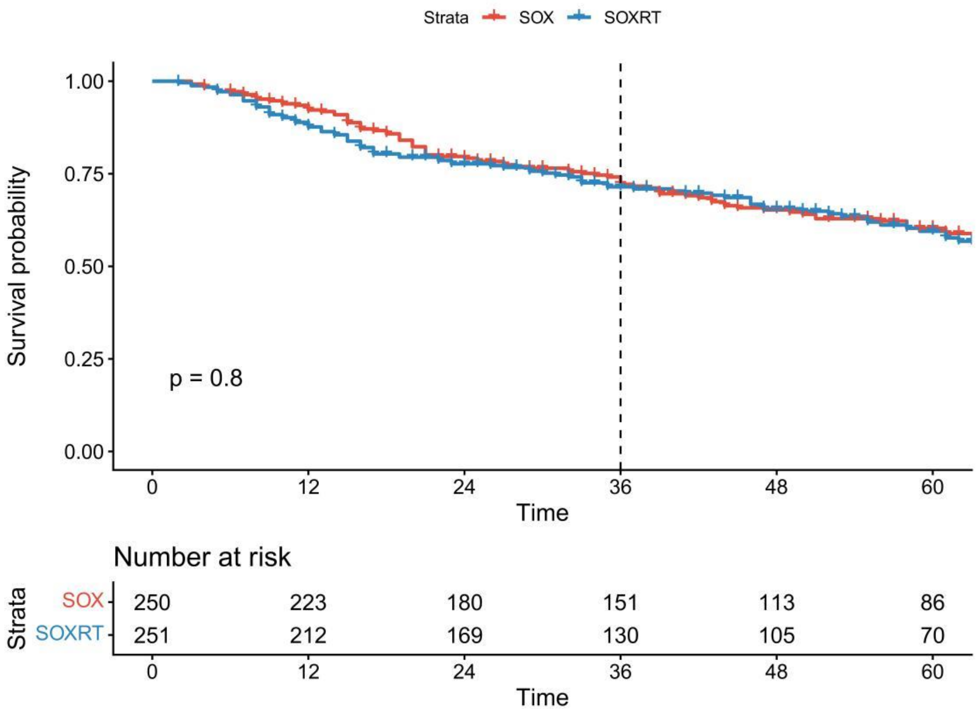

Kaplan-Meier curves for DFS in the per-protocol population. Curves are shown for SOX (n=250) and SOXRT (n=251). Tick marks indicate censored observations. The dashed vertical line indicates 36 months. Numbers at risk are shown below the x-axis. P=.80; P value was calculated using a 2-sided log-rank test. SOX indicates S-1 plus oxaliplatin; SOXRT, SOX plus radiotherapy.

© 2026 Wang X et al. *JAMA Network Open*

eFigure 3. Per-Protocol Overall Survival

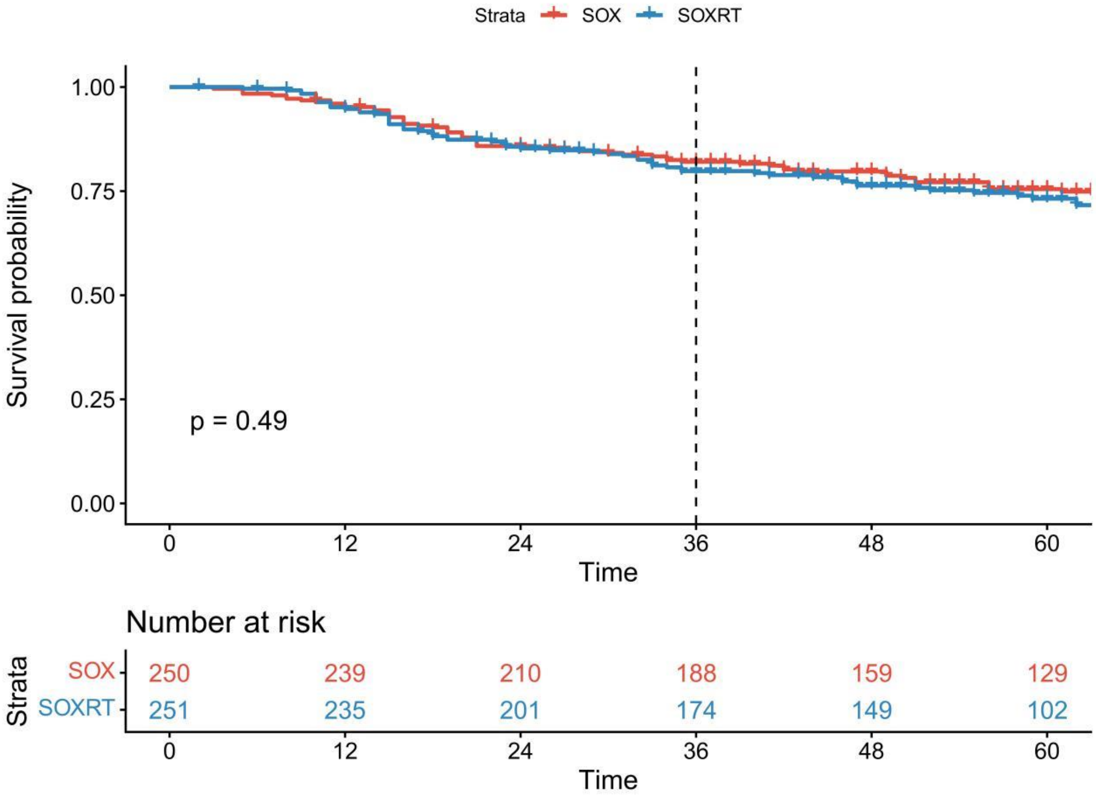

Kaplan-Meier curves for OS in the per-protocol population. Curves are shown for SOX (n=250) and SOXRT (n=251). Tick marks indicate censored observations. The dashed vertical line indicates 36 months. Numbers at risk are shown below the x-axis. P=.49; P value was calculated using a 2-sided log-rank test. SOX indicates S-1 plus oxaliplatin; SOXRT, SOX plus radiotherapy.

© 2026 Wang X et al. *JAMA Network Open*

eFigure 4. Subgroup Analyses of Overall Survival

Subgroup	Count	Percent	Hazard Ratio for OS (95% CI)	P for Interaction
**Overall**	620	100.0	0.91 (0.67, 1.24)	
**Sex**				0.609
Male	401	64.7	0.85 (0.59, 1.24)	
Female	219	35.3	1.02 (0.60, 1.76)	
**Age**				0.789
≤ 60	437	70.5	0.96 (0.66, 1.40)	
> 60	183	29.5	0.89 (0.52, 1.52)	
**Tumor site**				0.869
Cardia	90	14.5	0.99 (0.48, 2.02)	
Gastric body	209	33.7	0.84 (0.49, 1.44)	
Gastric antrum	310	50.0	0.90 (0.57, 1.41)	
Whole stomach	11	1.8	1.66 (0.17, 15.93)	
**Tumor size**				0.844
≤ 5 cm	354	57.1	0.89 (0.56, 1.43)	
> 5 cm	266	42.9	0.95 (0.63, 1.42)	
**Lauren**				0.430
Diffuse	302	48.7	1.13 (0.75, 1.70)	
Intestinal	168	27.1	0.79 (0.44, 1.40)	
Mixed	150	24.2	0.70 (0.31, 1.59)	
**Positive lymph node ratio**				0.465
≤ 25%	373	60.2	1.05 (0.66, 1.67)	
> 25%	247	39.8	0.83 (0.55, 1.24)	
**Lymphovascular invasion**				0.776
No	220	35.5	0.90 (0.53, 1.53)	
Yes	275	44.4	0.82 (0.54, 1.27)	
Unknown	125	20.2	1.17 (0.54, 2.53)	
**Perineural invasion**				0.209
No	181	29.2	1.15 (0.64, 2.05)	
Yes	358	57.7	0.72 (0.49, 1.07)	
Unknown	81	13.1	1.55 (0.60, 4.03)	
**AJCC stage**				0.503
I	24	3.9	1.51 (0.21, 10.72)	
II	160	25.8	1.43 (0.53, 3.84)	
IIIA−B	274	44.2	0.93 (0.60, 1.44)	
IIIC	162	26.1	0.68 (0.41, 1.12)	
**T stage**				0.114
T4a	263	42.4	0.65 (0.43, 0.98)	
T4b	11	1.8	1.65 (0.23, 12.05)	
Non−T4	346	55.8	1.23 (0.77, 1.98)	
**N stage**				0.207
N0	30	4.8	NA (NA, NA)	
N1	115	18.5	0.59 (0.23, 1.51)	
N2	192	31.0	0.85 (0.45, 1.62)	
N3	283	45.6	0.98 (0.66, 1.43)	

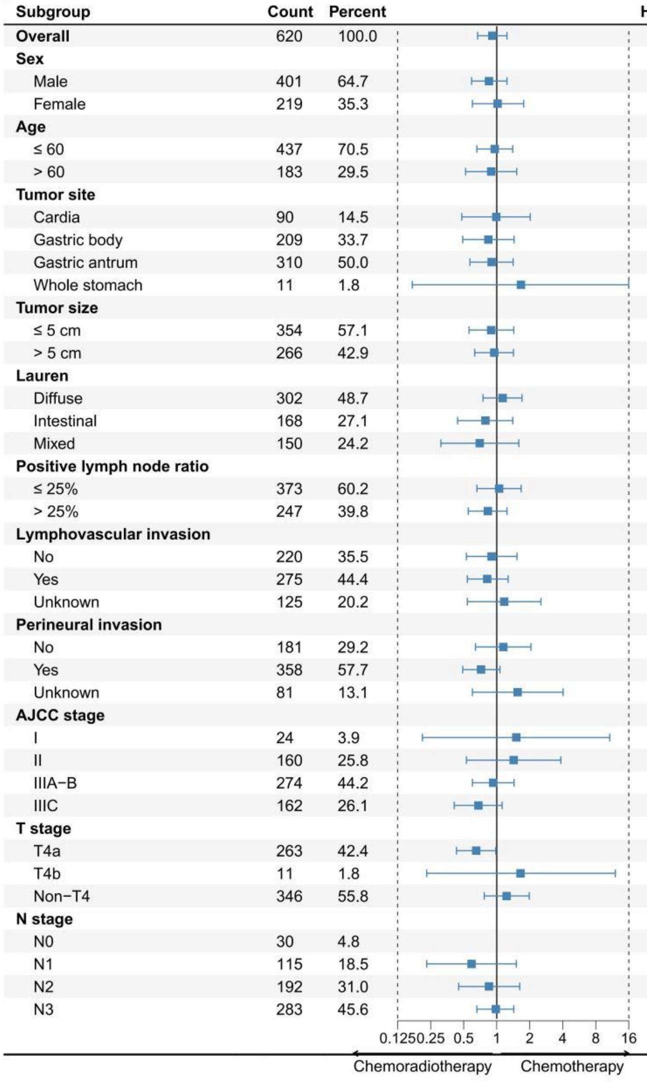

Hazard ratios (HRs) and 95% CIs were estimated using unadjusted Cox proportional hazards models within each subgroup (treatment group as the only covariate). P values for interaction were derived from Cox models that included a treatment-by-subgroup interaction term. HRs less than 1.0 indicate lower risk of death with SOXRT compared with SOX.

© 2026 Wang X et al. *JAMA Network Open*
