# Supplementary material for: Adjuvant Chemoradiotherapy or Chemotherapy After D2 Gastrectomy in Gastric Cancer: A Randomized Clinical Trial
Source: JAMA Netw Open. 2026 Jun 15;9(6):e2616154. doi: 10.1001/jamanetworkopen.2026.16154 (PMC13270272; doi:10.1001/jamanetworkopen.2026.16154)
Supplement: Supplement 3. — Data Sharing Statement [file jamanetwopen-e2616154-s003.pdf]

Data Sharing Statement

Wang. Adjuvant Chemoradiotherapy or Chemotherapy After D2 Gastrectomy in Gastric Cancer. *JAMA Netw Open*. Published June 15, 2026.
doi:10.1001/jamanetworkopen.2026.16154

Data
Additional Information: This trial was registered at ClinicalTrials.gov (ChiCTR-TRC-12002919).
Data available: No
